# Spanish Healthcare Sector Management in the COVID-19 Crisis Under the Perspective of Austrian Economics and New-Institutional Economics

**DOI:** 10.3389/fpubh.2022.801525

**Published:** 2022-03-18

**Authors:** Antonio Sánchez-Bayón, Esther González-Arnedo, Ángel Andreu-Escario

**Affiliations:** ^1^Applied Economics II Department, Legal and Social Sciences School, Universidad Rey Juan Carlos, Madrid, Spain; ^2^Business and HP Department, EAE Business School, Madrid, Spain

**Keywords:** healthcare management, accountability, transparency, reputation, communication, COVID-19, wellbeing economics, political economy, B5, D6, H5, I1, I31, K3, P16, P48, Z1.

## Abstract

This is a study of Political Economy, Law & Economics, and Public Choice, applied to COVID-19 crisis management, and how the Spanish healthcare sector has operated under stressful conditions. Market and state failures are evaluated and some improvements are offered, according to the theories of Austrian Economics and New-Institutional Economics. At the macro level, the premise is the decentralization of the Spanish healthcare system a long time ago, to provide a better service to citizens, according to the idiosyncrasies of the Autonomous Communities (similar to federal states). The crisis has evidenced the failures of the Spanish system and its semi-federal model, without coordination to manage the trouble. Also, the General Government's recentralization attempt has failed too, proving Mises's theorem on the impossibility of economic calculation in intervened and coactive systems, with problems of shortages, lack of coordination, etc.; Buchanan-Tullock's theorem on the unfinished agenda of state interventionist and it suppression of private sector was also proven. At the micro level, health institutions (hospitals and health centers) have fallen into the paradox of media overexposure and the fake-news risk, because the more information they have tried to transmit, the more confusion they have caused, reducing the value of the supposed transparency and accountability, in addition to decreasing citizen wellbeing, giving way to a higher level of dissatisfaction and more risk of a syndemic. To perform the analysis of accountability and wellbeing perceived, this paper has used quantitative contrast techniques on secondary sources, such as the surveys of *Centro de Investigaciones Sociológicas* (part of the Public Sector) or *Merco* rankings (independent institution).

## Introduction: Covid-19 Crisis, Its Management and Its Threats

In 1922, with the rise of the welfare economy and Government intervention in the economy (1, 2), Mises expressed his theorem about the impossibility of a centralized and enforced management in economy (as socialism presented), because it was against human action and its liberty to fix troubles and necessities (i.e., entrepreneurship, creativity, dynamic efficiency); also, this kind of management had negative results: bureaucracy, waste, corruption, lobbies, and rent-seekers (3–5). The unfinished agenda theorem by Buchanan and Tullock (6) completed the analysis about the interventionist state, which increases more and more, until it extend itself in the whole economy, without opportunities for the private sector. This paper aims to study the application of Mises' and Buchanan-Tullock theorems to the COVID-19 crisis management in Spain, under a socialist Government [also, it is a new realization of Peacock-Wiseman's hypothesis on public expenditure expansion during crisis (7–9)].

The current coronavirus, as a black swan (10), was recognized as a pandemic in March 2020 and was called SARS-CoV-2 or COVID-19. With many bad consequences, especially for health and the economy, it is necessary to mix management of both items, or there will be a syndemic risk (11). Also, the increased demand for information in times of health and economic crisis (12) requires bigger coordination and more accountability amongst state powers in the crisis management. This paper studies the Spanish case, with special attention to the healthcare sector, because there was mismanagement (of exceptionality abuse) in terms of health, economy, law, and accountability (13–15), with a decrease of wellbeing among the citizens (16), under the Austrian Economics approach (i.e., Mises' theorem, dynamic efficiency), and New-Institutional Economics (i.e., unfinished agenda theorem). How and why did it happen? The Spanish healthcare sector started the pandemic crisis from a precarious situation, due to cost reduction (17), less health-personnel (18), and structural problems (19) within the sector. Spain is neither a federal nor a centralized state; it is a regional system called an Autonomous country (20), which means different levels of resources and frameworks. The decentralization of the health system, a few decades ago, generated several scenarios among the regions or Autonomous communities (21). During the crisis, the Central Government aimed to recover the control of the healthcare sector, but the management of the crisis was wrong, because there was no common legal or political framework (21, 22). There were many mistakes on political economy (i.e., scarcity of health materials, non-coordination among the public sector and private sector, mass hysteria, and lockout), shown in studies in other papers (15, 23, 24). This paper focuses on the evaluation of the communication management and its coordination during the crisis way to prove a quality accountability and wellbeing for citizens. Also, it pays attention to the paradox that happened: as much information as was provided, there was more confusion, because of the non-coordination among the institutions involved in the healthcare sector, which had an inverse effect on the accountability and wellbeing perception by citizens.

Therefore, it is necessary to examine Spanish health management during the COVID-19 crisis, taking into account its regional decentralization, and using for that the tools of Austrian economics [i.e., Mises'theorem; (3, 4); reviewed by (25–35)], and new-institutional economics [i.e., Buchanan-Tullock theorem of unfinished agenda (5, 6); institutional quality: accountability, transparency and wellbeing analysis (32, 33); public choice: decision making, crony-capitalism risk & rent-seekers (5)].

This study is divided in to the following sections: first, the Spanish health system introduction, with attention to its special decentralized functionality and regulation. Second, the elements in relation: accountability analysis (communication and compliance mix, focused in transparency and reputation) and wellbeing evaluation (personal wellbeing related with communication and service offered by the health system). Third, the data and the methodology used to test the theorems (Mises and Buchanan-Tullock) and the secondary effects (i.e., mass-confusion increase: all information counter to the public communication is considered fake-news, which means more unwellness for the citizens). Fourth, the results obtained and a discussion on the accountability and the wellbeing evaluation of the health system in Spain during the pandemic is held.

### Healthcare System in Spain: Decentralized or Recentralized During the Pandemic?

The Spanish political-legal system is a semi-federal model: it is divided into 17 general regional governments or Autonomous Communities (ACs) and two special local governments or Autonomous Cities (20). The Spanish healthcare system is decentralized and transferred in several phases. The first step was the General Health Act of 1986, although the responsibility for health had already been transferred to Catalonia in 1981, followed by seven more Autonomous Communities and, finally, in 2001, it was extended to all of them. This means the coexistence of 17 health systems, one for each Autonomous Community, and the need for coordination of these systems by the Spanish Government (with its Ministry of Health), which maintains the provision of health services in the Autonomous Cities of Ceuta and Melilla (despite their Statutes of Autonomy of 1995).

The sections in the Spanish Constitution of 1978 (SC) included the possibility that the Autonomous Communities (ACs) could assume competencies in health according to a transfer process (section 148 and 150 of the EC). The General State Administration (State), for its part, would maintain the exclusive ability to establish the common bases and general coordination of healthcare throughout the Spanish territory (section 149 in SC), thus giving rise to an expansive, replicating, and asymmetrical Public Sector (34). Currently, there are almost 20 legal systems in Spain for ruling the Healthcare sector: this is not decentralization, it is a high cost of regulation (35).

The materialization of these provisions took place with the start of the decentralization process in health matters after the realization of the Autonomous Communities (1979–81). This process was asymmetrical among the different regions, both in the type of competencies assumed and in the time at which they were transferred (36). The so-called historical communities (Catalonia, the Basque Country, and Galicia), plus Andalusia, were the first regions to assume sanitary powers. The need for complete decentralization in this area (37) led to the extension of healthcare competencies to all the ACs (38, 39).

The nature of decentralization in health matters, which obeyed political rather than management and efficiency criteria (36), has led to the existence of a series of dysfunctions in the health system [manifesting itself in crises such as that of COVID-19 (23)]. These include the problem of health financing and spending (40), the disparity between ACs health systems (40), and the need for greater coordination (39). An example of this would be the inequality in access to health services among the ACs during the COVID-19 pandemic. This would be compounded by conflicts of competence in health matters between the national and regional levels of government (40). In Spain, the health problem does not have an economic base [because of the theorem on the impossibility of socialism (15)]; it is just a political issue (a game of power), so the communication and perception is a key point. For this reason, there would be an inverse relationship between communication and satisfaction (see later).

However, one of its benefits would be the capacity to adapt to the territory and the needs of the population (37, 38) and the increased capacity for transformation and innovation of the healthcare system (38, 41). In this way, decentralization would favor institutional change and improvement within the health system, as well as give more specific attention to citizens' demands. Consequently, addressing the Spanish health system implies taking into account each of the autonomous health systems, as well as the harmonizing role of the national government (to coordinate, not to centralize); in this way, it is possible to offer better economic policies (not just for healthcare).

Taking all of this into consideration, the accountability and evaluation of the Spanish health system during the 2019–2020 period is examined. This allows us to examine the consequences of health management before and during the COVID-19 pandemic, including the first two waves of it (and the emergency declaration passed for both cases).

### Accountability and Wellbeing Evaluation

The following point clarifies some of the key concepts analyzed in this study: on the one hand, accountability, which is comprised of transparency and reputation; on the other hand, wellbeing evaluation, which is comprised of personal wellbeing and communication about the health system.

#### Accountability in the Spanish Healthcare System: Transparency and Reputation Issues

Accountability, in terms of transparency, is an indispensable element in the framework of democratic systems. Citizens must have access to all the information surrounding political decision-making and the functioning of public services, so that they can exercise effective control over public authorities. However, information alone would not generate transparency, as it would require a complex process that would also depend on the context (42).

Transparency within the healthcare system is essential due to the need for accountability in an area as sensitive as healthcare. However, there are practically no studies that comparatively analyze the transparency of regional and national health systems (43).

Reputation reflects the external image of any organization. This reputation is shaped by the perception of different stakeholders (44, 45). In this sense, a large part of the reputation indicators are based on the perception of healthcare professionals and experts (45–47). However, it is pointed out that not only objective elements should be considered, but also subjective elements such as those related to user satisfaction (45). In this regard, citizen satisfaction would also be an important element of the efficiency of healthcare systems (48). For this reason, health care reputation indicators have also been constructed based on the perception of citizens and patients (49). In this sense, healthcare would be one of the public services best valued by citizens (40), despite its decentralization (15).

Hospitals are usually the most widely used healthcare organizations used to measure the reputation of the healthcare system (45, 46, 49, 50). The reputation of hospitals has a practical relevance, because it would condition users' preference for them (45). Accordingly, healthcare reputation cannot be examined without taking into account the decentralized nature of the Spanish healthcare system, since the characteristics of each Autonomous Community would affect hospital efficiency (46).

The problem on this topic happened during a crisis: what is the level of transparency in an emergency declaration? Can an alleged monopoly of information by the Government, declaring any criticism of its management as fake-news, be compatible with transparency? This could be the basis of mass hysteria (23, 24).

#### Wellbeing Evaluation of the Spanish Healthcare System

Due to the digital economy and in alignment with Horizon 2030, there is a shift from traditional welfare (social and material satisfaction) to wellbeing (personal and spiritual satisfaction also) (51, 52). This means that accountability is no longer heteronomous and limited to results (in accordance with state compliance regulations), but is becoming autonomous and people-oriented (companies are adopting internal codes for greater and better communication, participation and motivation of employees, and other stakeholders) (53). Wellbeing evaluation offers more satisfaction and goes beyond hygienic measures to include motivational ones (for workers, citizens, etc.). This paradigmatic shift means focusing on personal wellbeing, something especially important in a context of exceptionality and psychology, as the COVID-19 pandemic supposes. Therefore, it is of interest to examine the satisfaction of citizens with health management during this crisis, since the care received, or perceived, could make a difference in personal wellbeing.

The technologies of the digital economy facilitate the transformation and transparency in corporate culture around wellbeing evaluation (54, 55). Communication about the health system allows the transmission of management information to citizens, promoting personal wellbeing. New technologies, especially the Internet, and not just direct personal experience, can promote such communication without the mediation of traditional media. However, during the COVID-19 pandemic, paradoxically, instead of intensifying (as in other countries, e.g., South Korea, Taiwan, Israel, Australia, or New Zealand), in Spain it seems to have stagnated, resulting in public distrust, as evidenced by the reputational decline of the health sector (see later).

### Working Hypothesis and Premises Under Mises' Theorem and Buchanan-Tullock's Theorem

To research and discuss the hypotheses, we must start with the next question: How has the COVID-19 pandemic affected the Spanish health system in terms of accountability and wellbeing evaluation? Is it managed under a decentralized or recentralized system? The presumptions and presumed contradictions to analysis are: is it possible to come back to centralized management in a decentralized system during a pandemic and how does it help? Why, seeing as how much information was offered by the Healthcare institutions, was there more confusion among the citizens? Perhaps, does more intervention mean more security, accountability, and wellbeing or just the opposite and why? According to the theorems of Mises and Buchanan-Tullock, more intervention in a centralized and bureaucratic way means more control and scarcity, and this can be seen in material terms (e.g., there were not healthcare materials after the emergency declaration and the control of production and prices), but is this economic principle also applicable to immaterial issues such as information, accountability, and wellbeing?

## Materials and Methods

The methodology used in this research is mixed: (a) the theoretical framework of the Austrian Economics (56–61), and the New Institutional Economics (5, 6, 35); (b) the applied economics in COVID-19 crisis (62–64), related to the Spanish healthcare system (16); based on a quantitative approach using both descriptive analysis and bi-variable analysis, which allow observing the territorial differences among the variables for this study. The configuration of the Spanish healthcare system requires the research to be carried out from a regional perspective, that is, to examine the different elements of health management in each of the autonomous health systems, including those of national government management when necessary. In line with this, and in accordance with the hypotheses put forward, four variables to be examined in the decentralized Spanish health system are identified. Accountability of the health system encompasses the first two variables and wellbeing evaluation of the health system the last two variables.

The first variable is the transparency of the health system. This variable is measured by means of the transparency index of the autonomous health services carried out by Dynamic Transparency Index-Dyntra (65). This indicator is made up of a total of 193 indicators divided into six groups: institutional transparency (53 indicators), public communication (20 indicators), citizen participation and collaboration (22 indicators), economic-financial transparency (25 indicators), service contracting (14 indicators), and healthcare transparency (59 indicators). These data will make it possible to examine the transparency of the regional healthcare systems before the arrival of the COVID-19 pandemic (dated in 2019).

Similarly, in order to analyze the transparency of the regional health systems during the management of the COVID-19 pandemic, as well as that of the central government's management in this respect, the transparency index on COVID-19, also carried out by Dyntra, is used for healthcare systems and its transparency (URL: https://www.dyntra.org/indices/salud/transparencia-sobre-el-covid-19/). This index (2020) is made up of 40 indicators distributed into four groups: transparency in health resources (10 indicators), transparency on infections (nine indicators), actions to mitigate the impact of COVID-19 (nine indicators), and economic transparency in the management of COVID-19 (12 indicators).

The second variable is the reputation of the health system. This variable is measured through two different and complementary perspectives. In this sense, the reputation of any organization, including healthcare organizations, is shaped by the perception of different stakeholders (15, 40). Healthcare reputation, in line with this, has traditionally been measured through different indicators based on the perception of professionals and experts in the field.

Therefore, firstly, healthcare reputation is analyzed through Merco's Healthcare Reputation Monitor-MRS (66). This indicator is constructed through the evaluation of various types of healthcare professionals (doctors, nurses, and hospital pharmacists), patient associations, journalists specializing in healthcare, and members of the healthcare administration. These actors evaluate different elements of the healthcare system, including hospitals. In this way, they create a ranking of the 100 best hospitals in Spain. Based on this ranking, this research calculates a health reputation indicator that measures the percentage of hospitals out of the total number of existing hospitals that are included in the 100 most reputable hospitals in Spain. This is carried out in each of the autonomous health systems, thus making it possible to observe and compare the reputation of each of them prior to COVID-19 and during the pandemic.

Secondly, healthcare reputation is examined through the perception of citizens, who are the users and potential users of the healthcare system. For this purpose, data from the *Centro de Investigaciones Sociológicas* (CIS) on the perception of Spanish citizens of the functioning of the healthcare system are used. Two CIS'polls support this research: (a) Study No 3259 (in Oct. 2019, before the pandemic warning): sample of 2,464 interviews and a margin error of ±2.0% (67); (b) Study No 3290 (in July 2020, during the pandemic second wave): sample of 2,926 interviews and a margin error of ±1.8% (68). In both polls, the main question was: “In your opinion, how well is the healthcare system working? It works very, quite, little, or not at all well.” In this sense, a comparison is made of the percentage of citizens who state that this functioning is very or fairly satisfactory in each Autonomous Community. This perception is compared before and during COVID-19, thus observing whether or not the reputation that citizen's attribute to the health system in each region has changed with the arrival of the pandemic.

The third variable is personal wellbeing with the healthcare management in three aspects: (a) health system, (b) management of the Spanish Government, and (c) management of the Government of their respective Autonomous Community. A detailed analysis of the evolution of the satisfaction with these three aspects during the COVID-19 management itself is carried out, due to the fact that it has gone through different moments and phases. In this way, the aim is to discover how the satisfaction of the health management as perceived by the citizens of each Autonomous Community has varied throughout the pandemic. In this sense, we examine the percentage of individuals who declare that their satisfaction with the health system, with the management of the Spanish Government, and with the management of the Government of their respective Autonomous Community during the health crisis has improved, specifically between the first and second waves. Two CIS' polls are used: (a) Study No 3285 (June 2020): with a simple of 937 interviews and a margin error of ±3.3% (69); (b) Study No 3298 (Oct. 2020): with a simple of 2,861 interviews and a margin error of ±1.9% (70).

Finally, the fourth variable is communication about the healthcare management. The aim of this variable is to analyze how the flow of information about the health system has evolved with the arrival of the pandemic. The purpose of this is to observe whether or not the flow of information received by citizens about healthcare and its management, normally the actors most distant from it, has increased after the eruption of COVID-19. This is particularly important, since communication capacity facilitates the transmission of information, which is essential for transparency, and contributes to shaping the perception of the different stakeholders, especially the public, about the different health organizations and, consequently, their reputation, and ultimately the wellbeing evaluation with healthcare management.

As indicators of the communication variable, CIS survey data are used on the media by citizens to inform themselves about the pandemic (Study No. 3277) and on the average time of exposure to this media before and after the arrival of COVID-19 (Study No. 3305). (a) Study No. 3277 (March, 2020): with a simple of 3,911 interviews and a margin error of ±1.6% (71); (b) Study No. 3305 (Dec. 2020): a simple of 2,084 interviews and a margin error of ±2.2% (72). In addition to the information received through the media, citizens can shape their satisfaction with the healthcare system by their direct experience with it. For this reason, the percentage of individuals in each Autonomous Community who required healthcare before and during COVID-19 is also included. The results are available in several polls: (a) Study No. 3281 (May 2020), with a sample of 3,800 interviews and a margin error of ±1.6% (73); (b) Study No. 3,303 (Dec. 2020), with a sample of 3,817 interviews and a margin error of ±1.6% (74).

The data from the CIS surveys are perfectly comparable to each other, as shown by the multiple studies carried out with the data from this organization, since the CIS uses the same sample design and the same sampling procedure in all its surveys.

## Results and Discussion

The test of the hypotheses and premises has several answers to the research question, shown here in two parts: On the one hand, the findings related to accountability of the healthcare system (transparency and reputation) in the context of the COVID-19 pandemic are presented. On the other hand, the findings related to wellbeing evaluation of the healthcare system (personal wellbeing and communication) in the context of the COVID-19 pandemic are presented.

### Accountability of the Healthcare System in the Context of the COVID-19 Pandemic

#### Transparency of the Healthcare System During COVID-19

**Figure 1:**
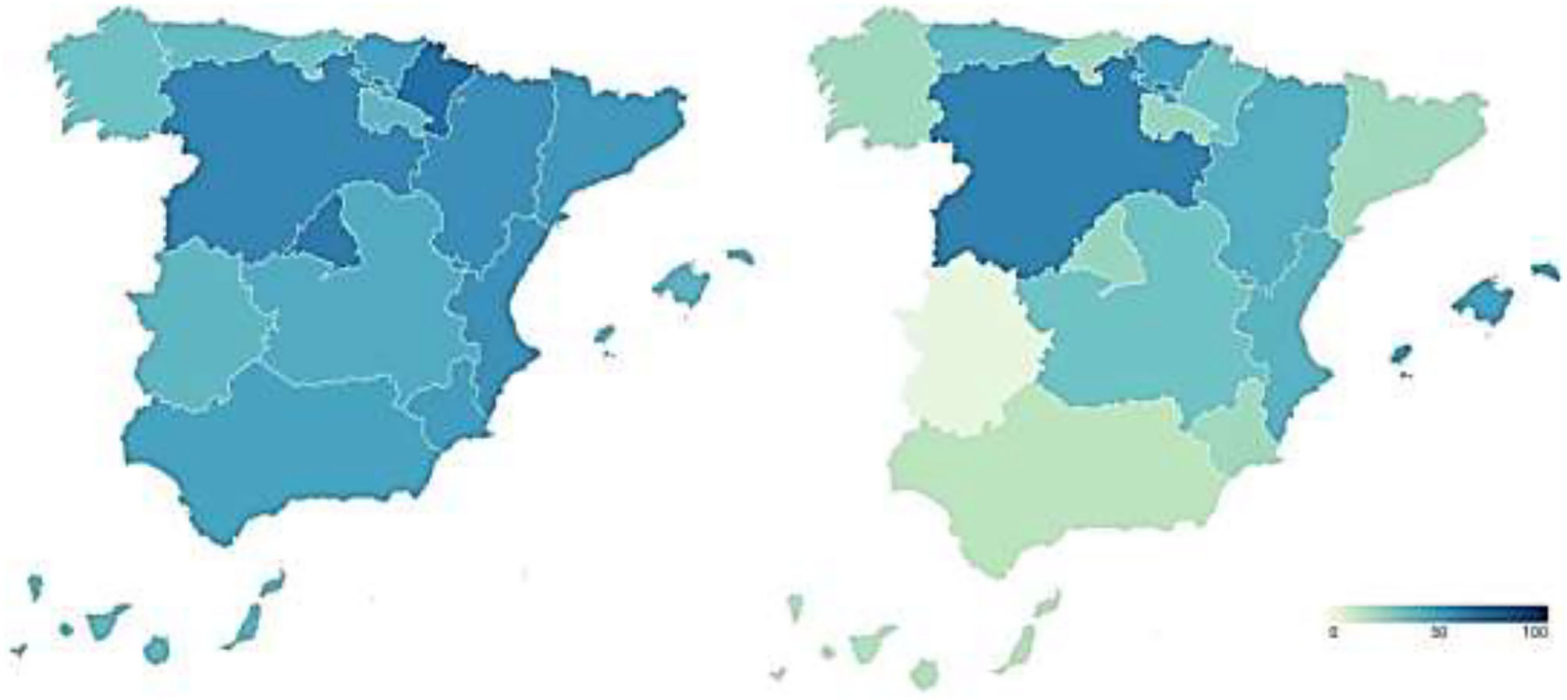
Transparency of Spanish regional healthcare systems before and during COVID-19 (%). Source: Peña-Ramos et al. (16).

To examine the transparency of health management during the covid-19 pandemic, transparency indicators before and during the pandemic are analyzed and compared. In this way, Figure 1 presents the transparency data of the regional health systems before (map on the left) and during (map on the right) COVID-19.

The transparency of the Spanish regional healthcare systems before the outbreak of COVID-19 is shown in the map on the left of Figure 1. It shows the percentage of transparency achieved by the healthcare services of each Autonomous Community in the index carried out by Dyntra. This percentage represents the number of indicators that each autonomous health system complies with out of the total number of indicators that make up the index.

Navarre was the region with the highest level of healthcare transparency before the arrival of the new SARS-CoV-2 coronavirus (70.47%). It was followed by the Community of Madrid (65.28%) and Castilla y León (60.62%). With percentages above 50% were Aragón (57.51%), the Community of Valencia (56.48%), Catalonia (53.89%), the Region of Murcia (51.81%), and the Basque Country (50.78%). The healthcare systems of Andalusia (49.74%), Castilla-La Mancha (46.63%), the Balearic Islands (41.97%), La Rioja (41.97%), Extremadura (41.45%), Asturias (39.38%), the Canary Islands (39.38%), Galicia (36.27%), and Cantabria (35.75%) failed in transparency. These regional differences in the transparency of the Healthcare system, previous to the pandemic, are relevant.

The transparency in the management of COVID-19 by the different Autonomous Regions is shown in the map on the right of Figure 1. This figure shows the percentage of transparency achieved by the healthcare management of the different Spanish regions during COVID-19 in the index carried out by Dyntra. This percentage represents the number of indicators that each Autonomous Community complies with out of the total number of indicators that make up the index.

Castilla y León is the Autonomous Community with the highest level of transparency in healthcare management during COVID-19 (62.5%). It is followed by the Basque Country (50%) and the Balearic Islands (50%), these three being the only regions to pass in transparency during the pandemic. The rest of the Autonomous Regions fail in transparency, with some of them showing particularly low levels. In this sense, Aragón (45%), the Valencian Community (42.5%), Asturias (40%), Navarra (35%), and Castilla-La Mancha (35%) have percentages between 45 and 35%. With scores below 30% in health transparency are La Rioja (25%), the Community of Madrid (25%), the Region of Murcia (22.5%), Galicia (22.5%), Catalonia (22.5%), and Cantabria (22.5%). The Canary Islands (17.5%) and Andalusia (15%) do not reach 20%, while Extremadura shows the lowest level of transparency in COVID-19 management with only 2.5%. On the other hand, the Government of Spain also shows a low level of transparency in COVID-19 health management (27.5%). Again, there are regional differences in transparency into the healthcare system, but this time, during the pandemic.

#### Reputation of the Healthcare System During COVID-19

**Figure 2:**
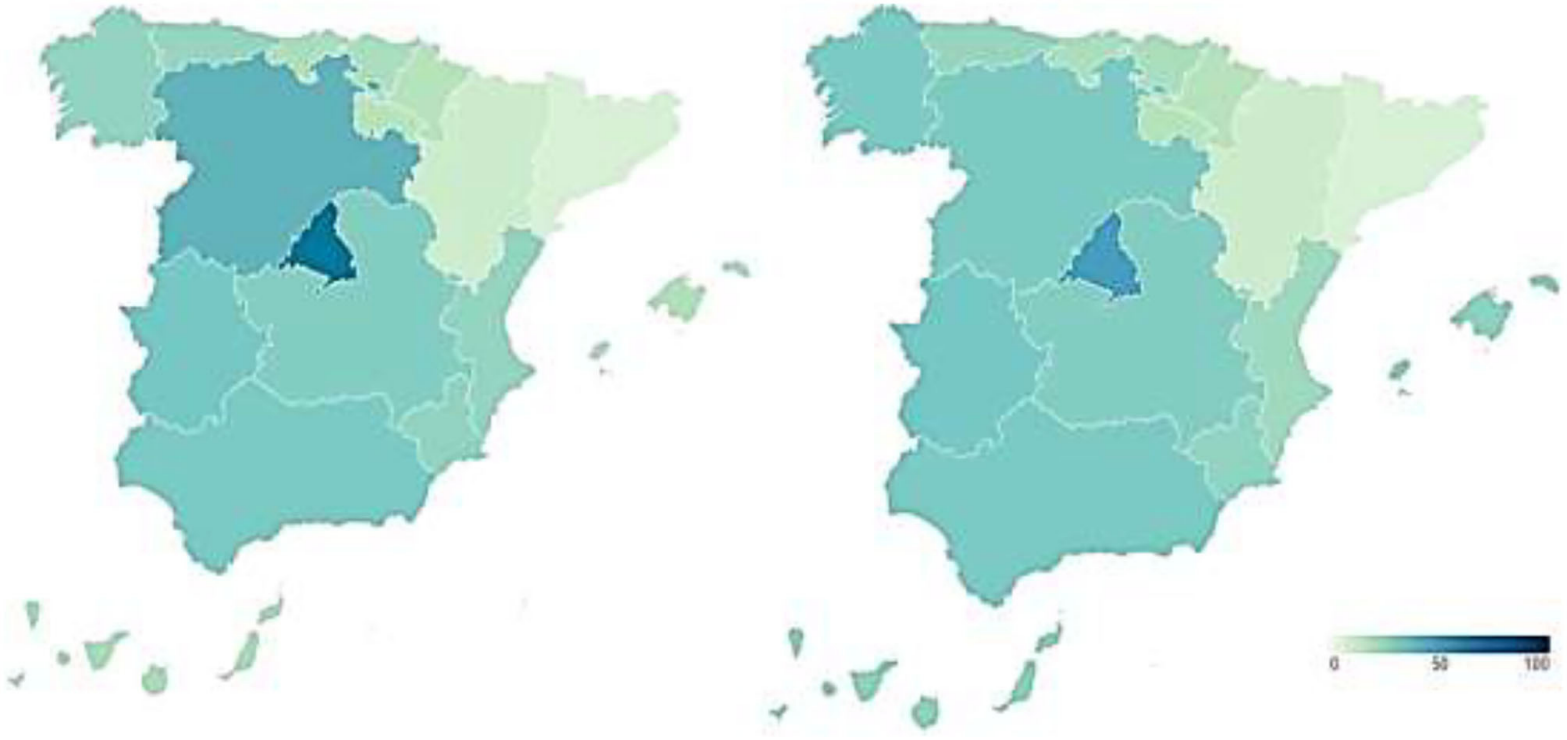
Reputation of Spanish regional healthcare systems before and during COVID-19 according to the perception of health professionals and experts (%). Source: Peña-Ramos et al. (16).

The reputation of the Spanish healthcare system is measured from two perspectives. Firstly, the reputation of the healthcare system is presented as a result of the perception of healthcare professionals and experts in the field. Figure 2 shows the percentage of hospitals in the top 100 of the Merco MRS out of the total number of hospitals that make up the healthcare system of each Autonomous Community before (map on the left) and during (map on the right) the pandemic.

As can be seen in the map on the left, the Community of Madrid stands out above the rest of the Autonomous Communities in terms of healthcare reputation, since 54.05% of its hospitals are among the top 100 hospitals in the ranking prepared by Merco before COVID-19. It is followed by Castilla y León (37.5%), Extremadura (33.33%), Andalusia (32%), and Castilla-La Mancha (30%). With levels below 30% in healthcare reputation are Murcia (26.67%), Galicia (26.32%), the Valencian Community (25.64%), Asturias (25%), the Canary Islands (20%), Cantabria (20%), and the Basque Country (20%). The regions with the lowest health care reputation are the Balearic Islands (18.18%), La Rioja (16.67%), Navarra (16.67%), Aragon (10%), and especially, Catalonia (6.96%).

As can be seen in the map on the right, the reputation of the regional healthcare systems, as a result of the perception of healthcare professionals and experts in the field, remains relatively stable with some changes in several Autonomous Communities. The Community of Madrid remains the region with the highest health reputation, although it experiences a slight drop to 51.35%. The Valencian Community and Castilla y León also experience a decline in the reputation of their health management during the pandemic, although of a moderate nature, falling to 23.08 and 31.25%, respectively. On the contrary, the Balearic Islands, the Canary Islands, and Galicia register an increase in their health reputation during COVID-19, by increasing the percentage of hospitals that are among the Top-100 in the country. These percentages are 27.27, 26.67, and 31.58%, respectively.

**Figure 3:**
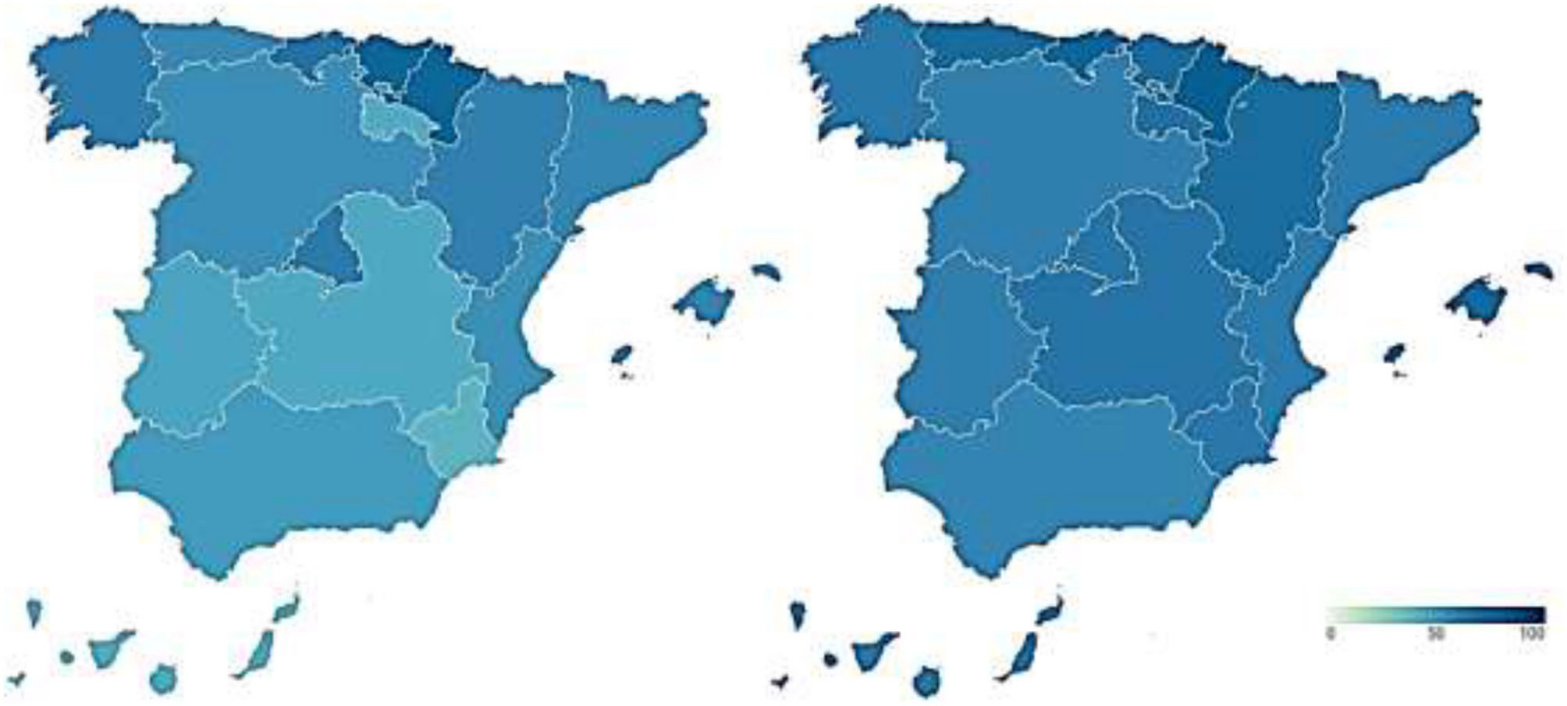
Reputation of Spanish regional health systems before and during COVID-19 according to citizen perception (%). Source: Peña-Ramos et al. (16).

Secondly, we incorporate the healthcare reputation resulting from the perception of Spanish citizens, who are the users and potential users of the healthcare system. Figure 3 shows these data broken down again by Autonomous Community, showing the percentage of citizens who say that the functioning of the healthcare system is very or fairly satisfactory in each of them before (map on the left) and during (map on the right) COVID-19.

Before the pandemic, the Basque Country (81.5%) and Navarre (81.3%) were the regions with the best healthcare reputation among their inhabitants. Cantabria (70.6%) also had a high level of citizen satisfaction with the functioning of its healthcare system, as did the Community of Madrid (65.6%), Galicia (64.8%), Aragon (63.4%), the Balearic Islands (61.8%), and Catalonia (61.3%). Similarly, more than half the population of Asturias (59.7%), Castile and Leon (58.2%), the Valencian Community (57.3%), and Andalusia (53.1%) had a positive image of the healthcare system in their Autonomous Community. In contrast, the healthcare systems of Extremadura (49.2%), Castilla-La Mancha (47.3%), La Rioja (47.1%), the Canary Islands (45.3%), and the Region of Murcia (42.5%) show a lower reputation among their citizens, although with not particularly low figures.

During the pandemic, the data show that the health crisis generated by the new SARS-CoV-2 coronavirus led to an increase in the reputation of the health system, according to the citizens' perspective, in all the Autonomous Communities 4 months after its outbreak. Thus, the percentage of citizens declaring themselves to be very or fairly satisfied with the functioning of the healthcare system is over 60% in all regions. The health systems of Navarre (84.6%) and Cantabria (82.1%) have the highest reputation among the population. They are followed by the Balearic Islands (78.1%), Asturias (77.4%), the Basque Country (76.6%), Aragon (75.9%), and La Rioja (73.3%). Lastly, we find Castilla-La Mancha (68.9%), the Community of Madrid (68.9%), Galicia (67.2%), the Region of Murcia (67.1%), the Canary Islands (66.9%), the Community of Valencia (65.8%), Catalonia (65.7%), Extremadura (64.4%), Castilla y León (63.7%), and Andalusia (61.7%). These data imply that there are no statistically significant differences between the reputations that citizens attribute to the healthcare systems of the different Autonomous Regions.

### Wellbeing Evaluation of the Healthcare System in the Context of the COVID-19 Pandemic

#### Personal Wellbeing With the Healthcare Management During COVID-19

Personal wellbeing with healthcare management is an essential issue, but it is especially so in a pandemic situation, due to the exceptional nature that it entails for all stakeholders. However, the management of the COVID-19 pandemic has gone through different moments and phases, so that citizen's perception of it may have varied accordingly, rather than being configured as a fixed image. In line with this, the following examines the satisfaction of citizens at two different moments of the health crisis on three different aspects: the health system, the management of the Spanish government, and the management of the government of their respective Autonomous Community. The percentage of citizens who recognize that their satisfaction with each of these has improved during the first wave (June 2020) and the second wave (October 2020) of the pandemic is represented.

**Figure 4:**
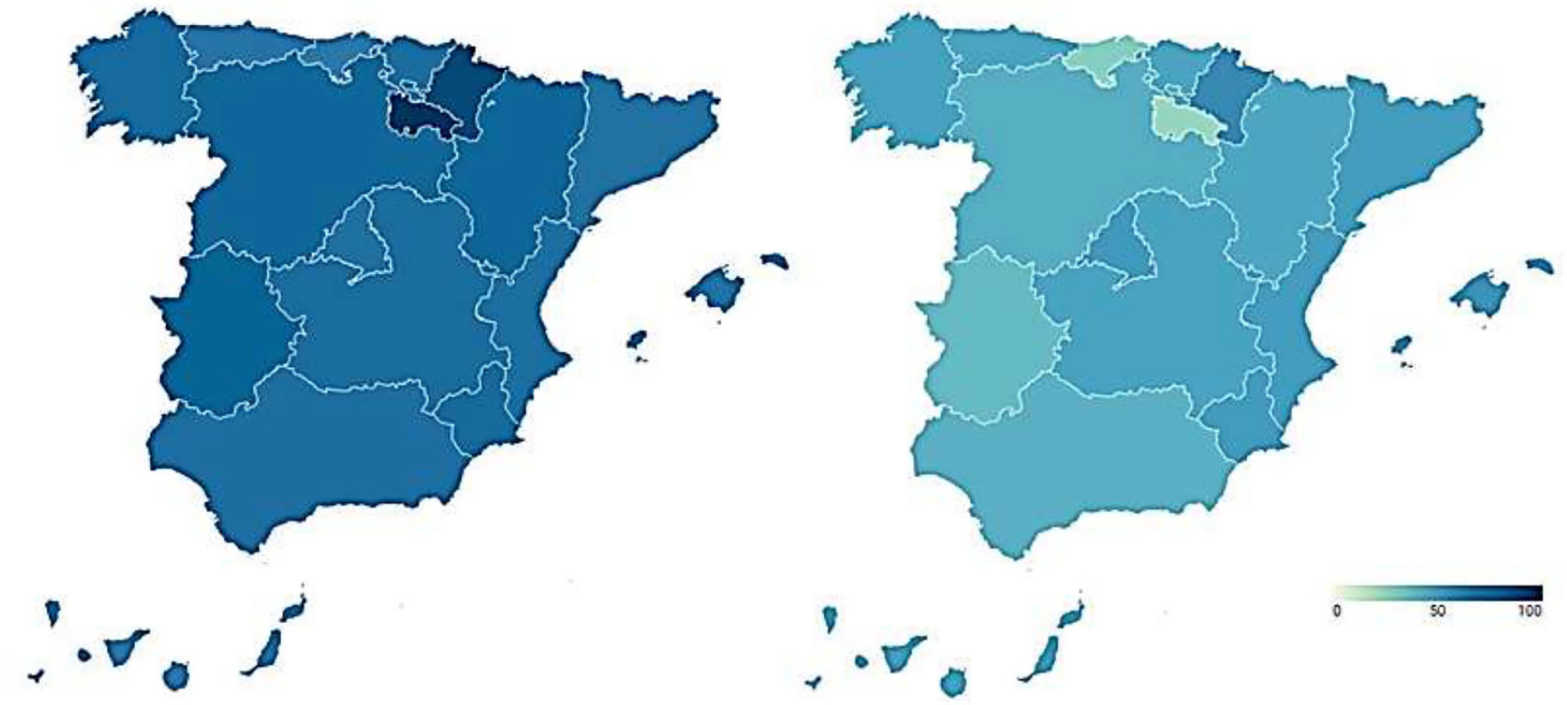
Citizen satisfaction with the health system during the COVID-19 1st and 2nd wave (%). Source: Peña-Ramos et al. (16).

Figure 4 presents the data on the improvement in citizens' satisfaction with the health care system during the first (map on the left) and second (map on the right) waves of COVID-19. The percentage of citizens indicating that their opinion of the health system has improved decreases over time. This means that citizen satisfaction with the health system has worsened over the course of the pandemic, despite being very positive at the beginning of the pandemic. La Rioja (75% points) and Extremadura (43.9% points) are the Autonomous Regions in which the proportion of citizens declaring that their image of the health system has improved has fallen the most. Next come Cantabria (36.9 points), Castile and Leon (35.7 points), Aragon (33.3 points), Navarre (32.3 points), and Andalusia (31.2 points). With decreases of <30% points are Galicia (27.4 points), the Basque Country (27.2 points), Castilla-La Mancha (26.6 points), the Region of Murcia (25.6 points), the Community of Valencia (21.9 points), Catalonia (20.4 points), and Asturias (20.3 points). The regions in which the satisfaction with the healthcare system has deteriorated the least are the Canary Islands (15 points), the Balearic Islands (15.4 points), and the Community of Madrid (19.7 points), in that order.

**Figure 5:**
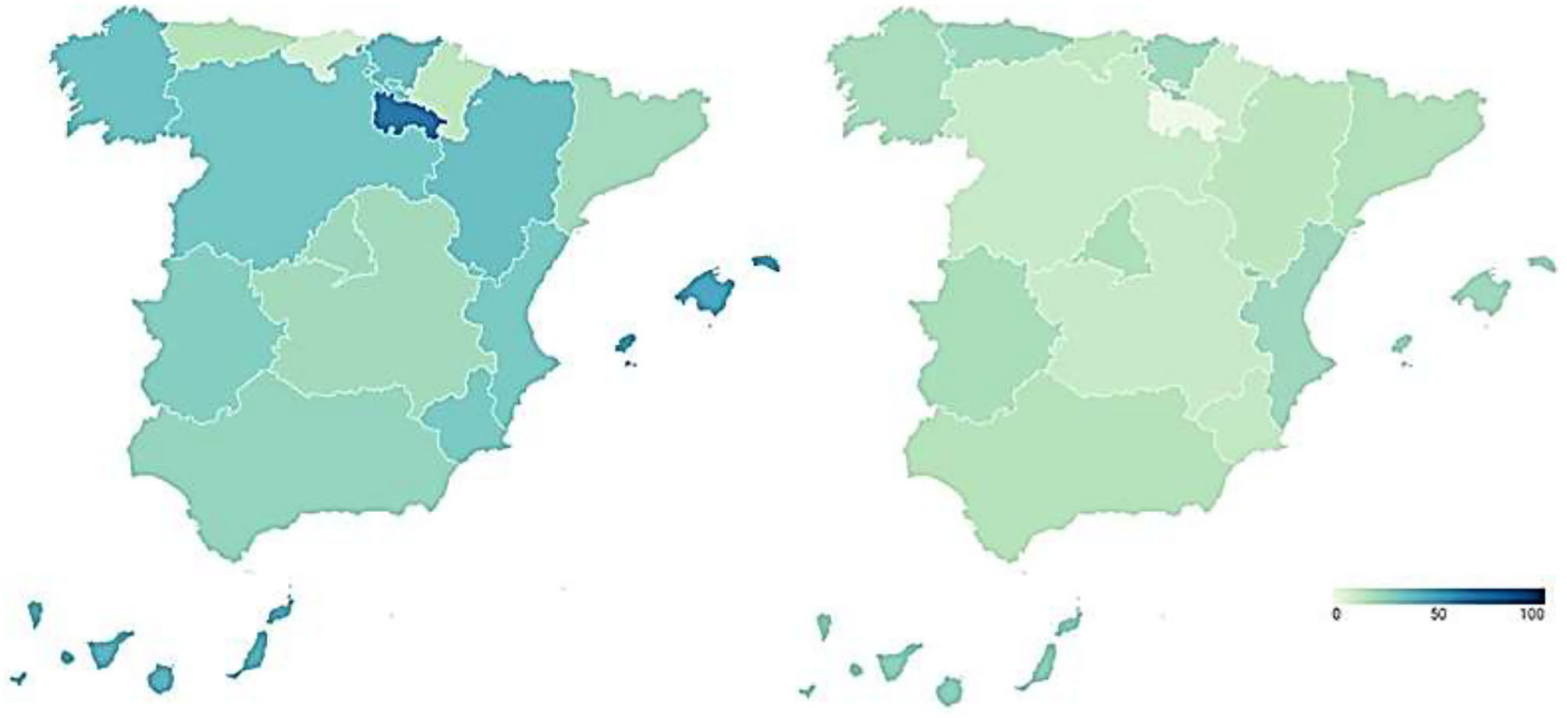
Citizen satisfaction with the management of the Spanish government during the COVID-19 1st and 2nd wave (%). Source: Peña-Ramos et al. (16).

Figure 5 shows the information on the improvement of citizen's satisfaction with the management of the Spanish government during the first (map on the left) and second (map on the right) waves of the pandemic. The percentage of Spanish citizens who say that their image of the national government has improved decreases throughout the health crisis. Thus, the satisfaction with the central government and its management of COVID-19 becomes more negative as time passes. However, the perception of the country's government was negative from the beginning of the pandemic, since only a small part of the population in most of the Autonomous Communities improved their satisfaction with it. Thus, the majority of citizens worsened their image of the central executive with the arrival of the COVID-19 pandemic. This negative perception also increased as the pandemic progressed.

The percentage of citizens who reported an improvement in their satisfaction with the Spanish government at the beginning of the health crisis did not exceed 40% in any region, with the exceptions of the Canary Islands (41.5%), the Balearic Islands (47.6%), and La Rioja (66.7%). This perception also worsened with the arrival of the second wave, with La Rioja, the Balearic Islands, Aragón, and Castilla y León being the Autonomous Regions most affected, with a drop of 66.7, 24.9, 22.2, and 21.6 percentage points, respectively. They are followed by the citizens of the Region of Murcia (18.9 points), Galicia (15.2 points), the Basque Country (15.2 points), the Canary Islands (14.3 points), and Castilla-La Mancha (10.3 points). Smaller differences exist between users in Extremadura (9.4 points), the Community of Valencia (9.3 points), Andalusia (8.9 points), the Community of Madrid (4.6 points), Navarre (4.3 points), and Catalonia (3.7 points). On the other hand, in Asturias and Cantabria the percentage of citizens who improve their satisfaction of the national government increased by 5.1 and 5.2 percentage points (each one).

**Figure 6:**
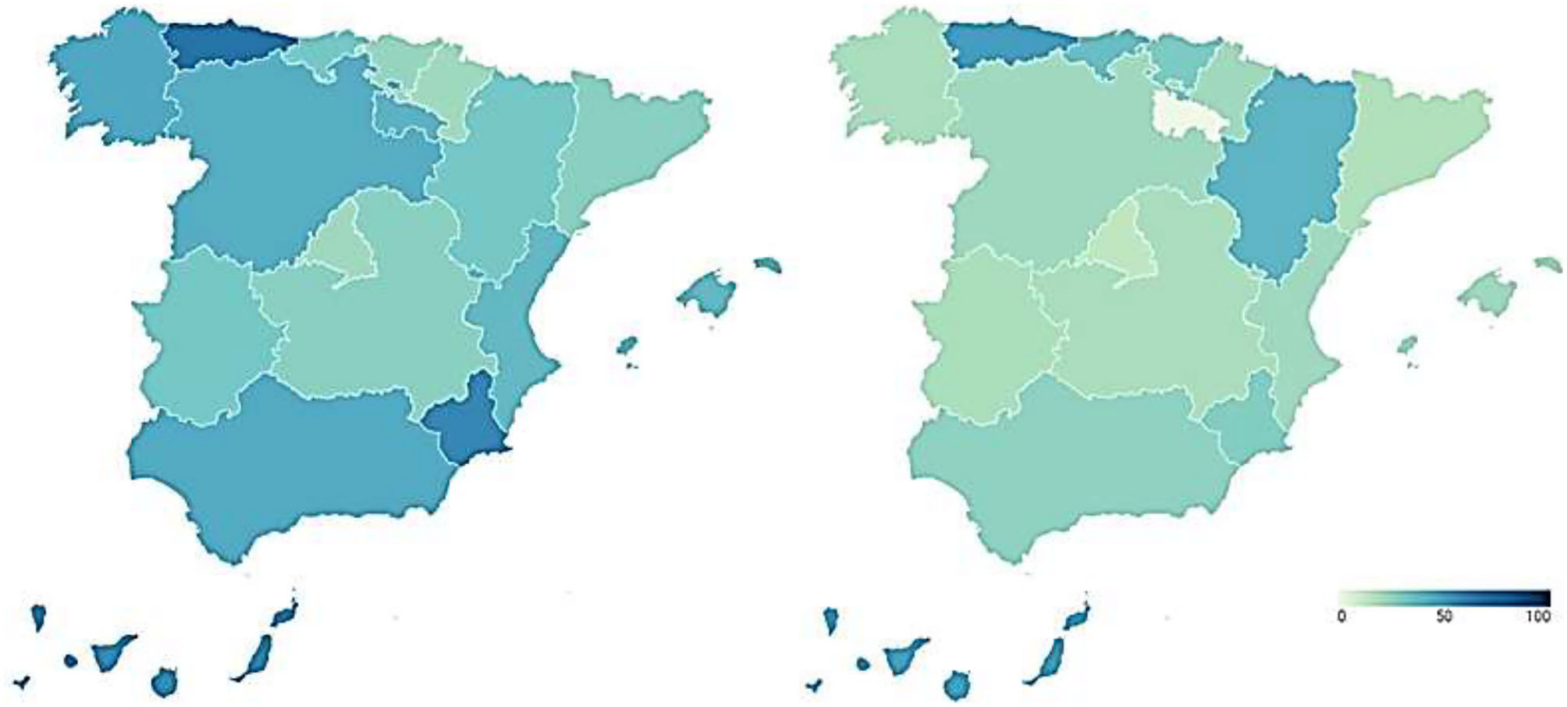
Citizen satisfaction with the management of the government of their respective Autonomous Community during the COVID-19 1st and 2nd wave (%). Source: Peña-Ramos et al. (16).

Finally, Figure 6 shows the data on the improvement of citizen satisfaction with management of the government of their respective Autonomous Community during the first (map on the left) and second (map on the right) waves of the pandemic. In the initial phase of the pandemic, there were disparities among citizens regarding the image of their respective regional governments. Thus, while in Asturias 69.6% of the population had improved their satisfaction with the regional executive, in Navarre this figure was only 23.1%. However, despite these differences, most of the Autonomous Regions experienced a worsening of citizen perception of the regional government throughout the pandemic, although in many cases this was not excessively high. Thus, this deterioration is more important in the Region of Murcia, amounting to 30.6% points. It is followed by Castilla y León (22.3 points), Galicia (20 points), Andalusia (19.3 points), Asturias (17.5 points), the Balearic Islands (17.3 points), and Catalonia (10.2 points). In the Community of Madrid (8.8 points), the Canary Islands (8.5 points), Castilla-La Mancha (8.4 points), Extremadura (6.6 points), and La Rioja (5.4 points), on the other hand, the change of opinion is smaller. Likewise, in Navarra (0.6 points) and in the Valencian Community (0.2 points), the satisfaction with the regional executive remained practically stable throughout the pandemic. On the other hand, the citizens of Cantabria, the Basque Country, and Aragon improved their image of the regional government between the first and second wave of the pandemic, with 4.5, 6.4, and 8.6% of them doing so. Thus, we can see that there are regional differences in the citizen satisfaction with their respective regional executives.

#### Communication About the Healthcare Management During COVID-19

Personal wellbeing with the health care system is conditioned by two elements: the image offered by the media and direct experience with it. Both factors contribute to shaping individuals' image of the healthcare system and, consequently, their satisfaction with it. They are also the instruments through which the health system and its managers can transmit information on its functioning, thus increasing its transparency and reputation.

**Figure 7:**
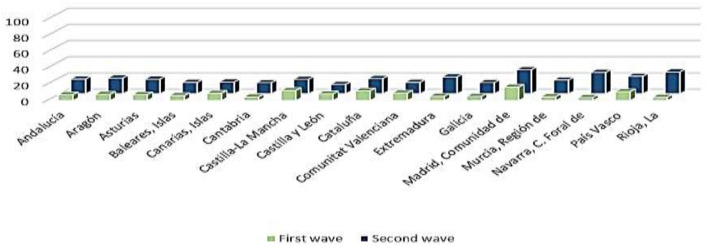
Citizens' direct experience with healthcare services during the COVID-19 1st and 2nd wave (%). Source: Peña-Ramos et al. (16).

As a consequence, both the direct experience of citizens with the health system and their exposure to the media during the pandemic are examined. Thus, Figure 7 shows the percentage of citizens who have visited the health services for symptoms related to the coronavirus during the first and second waves of the pandemic. These data are broken down by Autonomous Community. The proportion of citizens who have had a direct experience with health services does not exceed 30% in any of the pandemic waves considered. During the first wave of COVID-19, the citizens of the Community of Madrid (16.4%), Castilla-La Mancha (12.2%), Catalonia (11.8%), and the Basque Country (10.8%) were those who had to resort to the health system to the greatest extent, although they represent a very small percentage of the population. During the second wave, the Community of Madrid (29.6%), La Rioja (26.9%), Navarra (26.4%), the Basque Country (21.7%), and Extremadura (20.8%) were the regions that recorded the greatest contact of their citizens with health services, also representing limited proportions of the population. Consequently, the majority of citizens had no direct contact with the health system during the first two waves of the pandemic.

**Figure 8:**
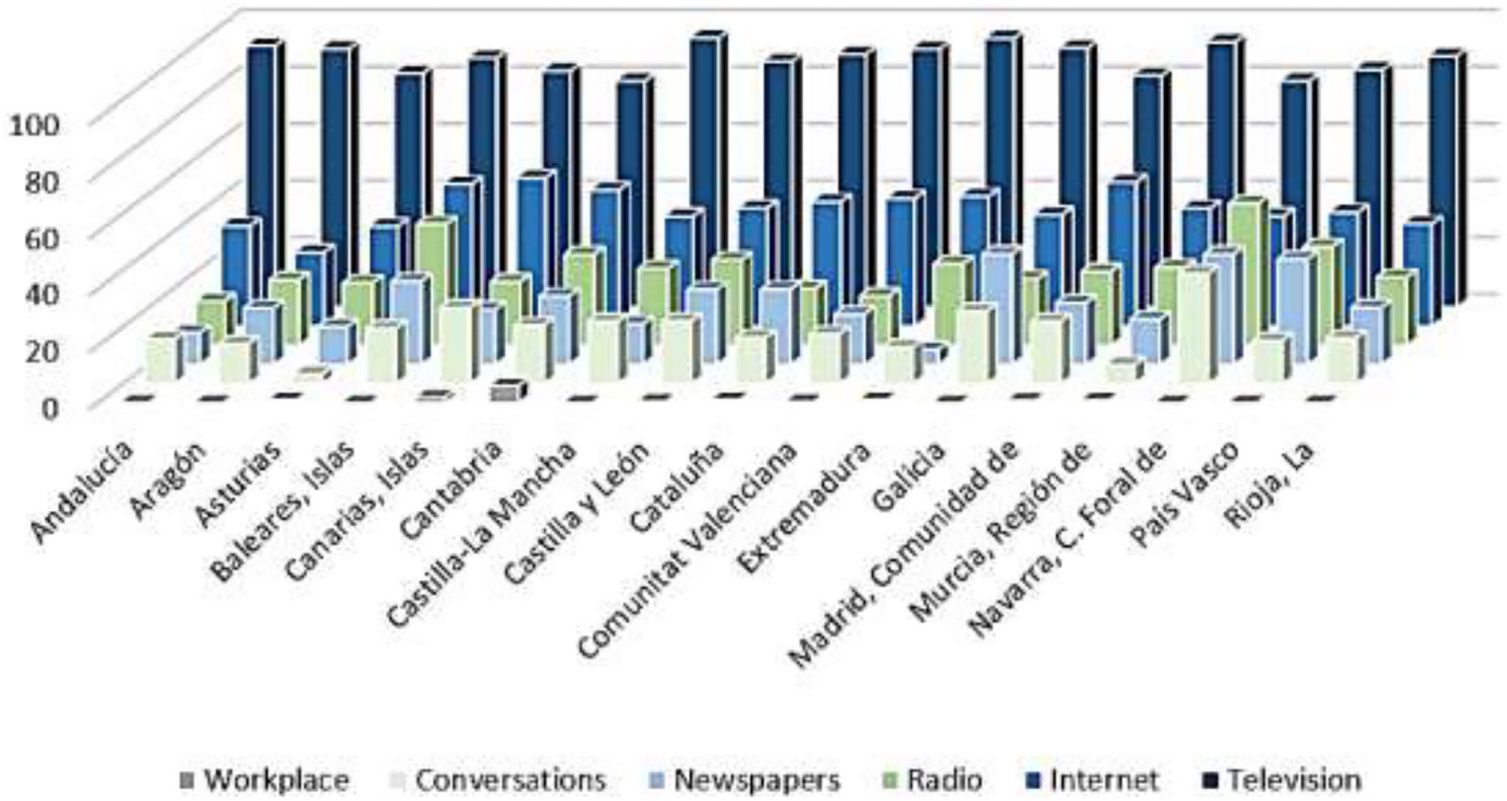
Media most used by citizens to obtain information on the COVID-19 pandemic (%). Source: Peña-Ramos et al. (16).

This implies that a large part of the citizenship obtains information about the health system, and especially about the management of the pandemic, through the media. Thus, they would largely shape their perception of the health system and the management of COVID-19 through the media. In relation to this, more than 90% of Spanish citizens have followed the news related to the coronavirus with some interest. Figure 8 shows the media through which they have followed the news about the pandemic. Television is configured as the main means of information for Spanish people during the health crisis. More than 8 out of 10 citizens used it to follow COVID-19 news in all the ACs. Internet is the second most used channel, either through social networks, digital press, or any other online media. Around 4 out of 10 Spanish citizens were informed about the pandemic through the Internet, with regions where this figure is even higher: the Canary Islands (52.2%), the Community of Madrid (50.7%), and the Balearic Islands (50%). Radio is the third most used means of communication. More than 2 out of 10 Spanish citizens have heard news about COVID-19 on the radio, with more than 4 out of 10 in regions such as the Balearic Islands. Close behind is the written press, followed by obtaining information in conversations on the subject. The workplace, on the other hand, is a very residual medium for information on the pandemic.

**Figure 9:**
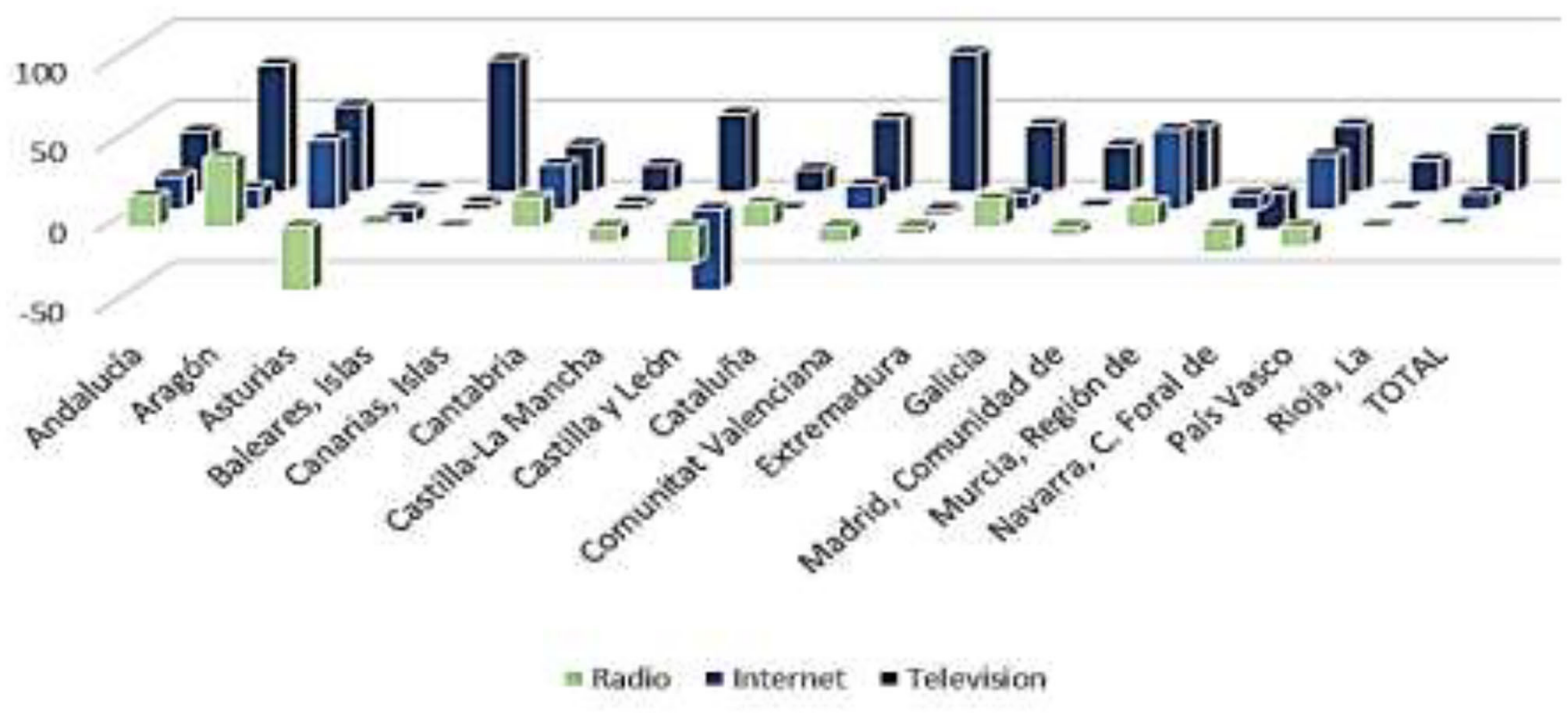
Average increase in time of exposure to the main media before and during COVID-19 (minutes). Source: Peña-Ramos et al. (16).

Having identified the three forms of media through which citizens have been most informed about the health crisis, it is then observed whether exposure to them has increased or not during the pandemic. As shown in Figure 9, the time of exposure to all media increased during the pandemic. Television is the medium whose use has increased the most. Thus, Spanish people watch 38.0 min more television per day on average than before the arrival of COVID-19. The use of social networks, specifically through the Internet, has also increased. Thus, citizens have increased by 9.9 min on average the daily time they spend connected to social networks. Likewise, although to a lesser extent, the time Spanish citizens spend listening to the radio has also increased. Thus, they are exposed to this medium for an average of 1.5 min more per day. Therefore, it is confirmed that the demand for information increases in times of health crisis and it has an impact in satisfaction, as there are relevant differences in media exposure after the arrival of the pandemic.

Taking into account regional differences, it is necessary to point out that the Autonomous Communities in which television consumption grew the most were Extremadura (86.4 min), the Canary Islands (82.2 min), and Aragon (79.2 min). The regions that most increased their exposure to the Internet were the Region of Murcia (49.2 min), Asturias (43.2 min), and the Basque Country (33.6 min). Finally, radio consumption increased especially in Aragon (42.6 min), Andalusia (18.6 min), and Cantabria (18.0 min).

However, despite the increase in average consumption of the media, some regions experienced decreases in some of them during the pandemic, especially in radio (Asturias, the Canary Islands, Castilla-La Mancha, Castilla y León, the Valencian Community, Extremadura, the Community of Madrid, Navarra, and the Basque Country) and, to a lesser extent, on the Internet (the Balearic Islands, Castilla y León, Catalonia, and Extremadura). Exposure to television, for its part, only decreased in Navarra, confirming this as the main media through which citizens were informed of the health management of COVID-19.

#### Discussion and Implications

This research has examined the reputational and wellbeing consequences of the COVID-19 pandemic on health management in Spain, under a Socialist Government, which presented the recentralization of powers during the crisis, with the excuse of a higher and more equal protection. This research has studied the development of two key elements for healthcare management before and during the arrival of the new SARS-CoV-2 coronavirus: accountability (transparency and reputation) and wellbeing evaluation (personal wellbeing and communication). Due to the decentralized system in Spain, this analysis is carried out by disaggregating the data by Autonomous Community (20, 34). The results show that the health crisis triggered by the arrival of the COVID-19 pandemic has had a considerable impact on health management, producing changes in all the elements analyzed. In general terms, it can be stated, firstly, that there has been a deterioration in the transparency of the Spanish health system during the health crisis. From a situation of relatively acceptable transparency in most regions, although with significant differences between them, the situation has shifted to one of greater opacity. Public information on the management of the pandemic is limited in all the Autonomous Regions, as well as on the part of the national government, considerably increasing the disparities between regions. In this sense, the public authorities have had to transform their communication policy, based on traditional information on health services, to a totally different reality, that of a new and unknown pandemic disease, having to do so in conditions of health emergency, which has shown a significant deficiency in respect of such management.

Related with the reputation of the healthcare system is the perception of various stakeholders (15, 40). This article has analyzed it from the perspective of professionals and experts in the field, on the one hand, and from the perspective of citizens on the other. The health reputation from the perspective of health professionals and experts shows the existence of great divergences between Autonomous Regions before the arrival of the pandemic. With the arrival of the health crisis the data on health reputation from this perspective does not show important changes. Some regions slightly worsen their health reputation, while others improve it moderately. Healthcare reputation from the citizen's perspective, on the other hand, presents more positive data, although regional differences are maintained, which are reduced until they practically disappear during COVID-19, as homogeneity in the perception of regional health care systems increases. In relation to this, the arrival of the pandemic brought with it an increase in the reputation of the health system from the public's perspective.

The subsequent development of the pandemic led to a deterioration in personal wellbeing with healthcare management during COVID-19. This deterioration occurs especially with respect to the health management of the national government and, after it, with respect to the health management of the governments of the Autonomous Communities. Likewise, the image of the health system is also undermined.

Finally, with regard to communication, there has been an increase in the flow of health information with the COVID-19 arrival; at the same time, there was an increase of confusion, not just for the contradictions between the information transmitters (at least 20 officials powers), changing each time, but also for the insinuation of fake news to all information contrary to the official version of the National Government.

At the beginning of the COVID-19 crisis, the majority of citizens followed the news about the pandemic with interest. Likewise, there has been an increase in the amount of time that citizens have been exposed to the most widely used media to learn about the health crisis. This implies that information on healthcare and its management is not only more in demand, but also floods the media. Therefore, the visibility of healthcare management has increased enormously. In this way, Hypothesis 4 is accepted. Nevertheless, the fact that most citizens have not had direct contact with the healthcare system means that they have access to information, which is essential for transparency, shaping their perception of the healthcare system, and consequently the reputation of healthcare, through the media. This may help explain the deterioration in personal wellbeing with health management during the pandemic because it could have showed as a problem of perception and infoxication (information overload and contradictions between the transmitters).

Previous studies on healthcare management and its different elements during crisis (64), with special attention to accountability and wellbeing evaluation (16), have not paid attention to the theorems of Mises and Buchanan-Tullock applied to crisis management. However, the outbreak of a pandemic such as the one that occurred with COVID-19 poses a main challenge for research in this area. The healthcare system is on the front line in the fight against the new SARS-CoV-2 coronavirus, so it is essential to examine how the pandemic has affected it not only internally, but also in its external projection. Therefore, accountability and wellbeing evaluation of healthcare management requires an in-depth analysis in this context. This not only addresses the perspective of professionals and experts in the field, but also the citizen's perspective, which is unavoidable in this scenario. Therefore, the relationship of citizens with the health system and its managers at different levels of government is taken into consideration.

This research is a first step in the study of the effects of COVID-19 on health management, reaching important conclusions. The information obtained may be valid not only for accountability, but also for decision making by public authorities regarding the different elements of health management. This is also done in a case such as Spain, which has been one of the European countries most affected by the pandemic (as has been officially recognized).

This research has several limitations derived from the timing of its implementation. In this regard, by examining the consequences of the pandemic on health management while still in the midst of the pandemic, we do not yet have a sufficient amount of data to allow a more in-depth analysis of the two elements of health management under investigation: accountability and wellbeing evaluation. Likewise, with respect to the study period, we have only been able to investigate the consequences of the COVID-19 pandemic on Spanish health management during the first two waves of the pandemic.

Nevertheless, this research is a first approach that may contribute to future research to advance the study of health management in terms of accountability and wellbeing evaluation once the pandemic is over. In this way, broad conclusions can be reached on the overall effect of COVID-19 on the external projection of the healthcare system. In this respect, the approach adopted in this research, disaggregating the data by region due to the decentralized nature of the Spanish health system, may prove useful.

The impact of the stoppage on the transformation of the welfare model remains to be studied in depth (in its shift to wellbeing model), as the company has not relied on the mobile technologies of the digital economy during its management (11). This has caused not only a setback in motivational measures, but also hygienic measures of the affected collaborators. For the possible development of this line, sources such as Great Place To Work (GPTW) and Organization for Economic Cooperation and Development (OECD) health reports, as well as other specialized complementary sources, will be used.

## Conclusions

The Spanish healthcare system and its communication management during the COVID-19 crisis was unsuccessful, especially for the non-coordination among the institutions in the public sector and with the private sector. There was a double management problem: a legal and political mismanagement (without clear coordination and regulation) and a worse communication management (with contradictions between the official transmitters, with bad perception about it—also, generating mass hysteria). There was an inverse relation between communication and satisfaction, because the more information was provided to the citizens, the worse satisfaction was obtained with the healthcare system, as a problem of perception and infoxication (information overload, fake news, and contradictions among public powers declarations). Also, this study shows that the healthcare management and the citizens' perception have changed during the crisis. Possibly, the situation of the healthcare system has been complicated because of the fuzzy decentralization in the Spanish semi-federal system: many competencies were transferred years ago, without a clear legal and political framework for coordination (the result was a healthcare monopoly for each Autonomous region). During the crisis, the Central Government pretended to re-centralize the competence in healthcare, but the confusion was bigger and non-functional (as the theorems of Mises and Buchanan-Tullock previewed). Also, it seems that the advances achieved in healthcare management of wellbeing (more satisfaction and better relations between people-planet-profit) has been rejected during the crisis, losing attention to improve satisfaction and motivational measures. This is a key point, because the COVID-19 crisis is not just a pandemic, it is also a potential syndemic (as a topic for future lines of research).

A corollary, linked with the unfinished agenda (Buchanan-Tullock's theorem), is the false clash between public and private services in the healthcare system. Part of the management by the Central Government was to support the public sector against the private companies (i.e., Central Government communicated that the public hospitals suffered scarcity of healthcare materials because the private hospital kept it—as unsupported business-). In this way, the Central Government promoted just the public sector in the distribution of new medical supplies, vaccines, etc., and the assignation of recovery funds. The result is the public choice problem: no voice, no exit (there is not possibility to critique and there is no alternative to receive healthcare services).

## Author Contributions

All authors listed have made a substantial, direct, and intellectual contribution to the work and approved it for publication.

## Funding

This research formed part of the Ph.D. dissertation in Economics, supported by GESCE-URJC, GID-TICTAC CCEESS-URJC, Centro de Doctorado Henry Hazlitt-UFM, and TRANS-REAL LAB-UVA.

## Conflict of Interest

The authors declare that the research was conducted in the absence of any commercial or financial relationships that could be construed as a potential conflict of interest.

## Publisher's Note

All claims expressed in this article are solely those of the authors and do not necessarily represent those of their affiliated organizations, or those of the publisher, the editors and the reviewers. Any product that may be evaluated in this article, or claim that may be made by its manufacturer, is not guaranteed or endorsed by the publisher.

## References

[1] Pigou AC. The Economics of Welfare. London: Macmillan (1920).

[2] Keynes JM. The General Theory of Employment, Interest and Money. London: Macmillan (1936).

[3] Mises L. Die Gemeinwirtschaft: Untersuchungen über den Sozialismus. Jena: Gustav Fischer Verlag (1922).

[4] Mises L. Kritik des Interventionismus: Untersuchungen zur Wirtschaftspolitik und Wirtschaftsideologie der Gegenwart. Jena: Gustav Fischer Verlag (1929).

[5] Buchanan J Tullock G. The Calculus of Consent: Logical Foundations of Constitutional Democracy. Ann Arbor, MI: The University of Michigan Press (1962).

[6] Anderson J. The Unfinished Agenda: Essays on the Political Economy of Government Policy in Honour of Arthur Seldon. London: Institute of Economics Affairs (1986).

[7] Peacock AT Wiseman J. The Growth of Public Expenditure in the United Kingdom. Princeton, NJ: Princeton University Press (1961).

[8] Peacock AT Wiseman J. Approaches to analysis of government expenditure growth. Publ Fin Q. (1979) 7:3–23. 10.1177/109114217900700101

[9] Henrekson M. Peacock and Wiseman's displacement effect: a reappraisal and a new test. Eur J Polit Econ. (1990) 6:245–60. 10.1016/0176-2680(90)90054-M

[10] Taleb N. The Black Swan. The Impact of the High Improbable. New York, NY: Random House (2007).

[11] Sánchez-Bayón A. Balance de la economía digital ante la singularidad tecnológica: cambios en el bienestar laboral y la cultura empresarial. Sociologí*a y Tecnociencia*. (2021) 11:53–80. 10.24197/st.Extra_2.2021.53-80

[12] Rodríguez JS. Periodismo, comunicación institucional y transparencia: aprendizajes de la crisis sanitaria del COVID-19. Rev Comun Salud. (2020) 10:569–91. 10.35669/rcys.2020.10(2).569-591

[13] Bueno L. Feer seen from law. Bajo Palabra. (2021) 27:307–22. 10.15366/bp2021.27.016

[14] Duque GA Del Prado C. Agamben is right: COVID 19 and permanent state of exception. Bajo Palabra. (2021) 27:105–24. 10.15366/bp2021.27.005

[15] Huerta de Soto J Bagus P Sánchez-Bayón A. COVID-19 crisis management and cost estimation models: bureaucratic government coaction vs. spontaneous social coordination. Preprints. (2021) 2021:2021050024. 10.20944/preprints202105.0024.v1

[16] Peña-Ramos JA Recuero-López F Sánchez-Bayón A Sastre FJ. Evaluation of spanish health system during the COVID-19 pandemic: accountability and wellbeing results. Int J Environ Res Public Health. (2021) 18:12907. 10.3390/ijerph182412907 34948518 PMC8701968

[17] Navarro V. El error de las políticas de austeridad, recortes incluidos, en la sanidad pública. Gaceta Sanitar. (2012) 26:174–175. 10.1016/j.gaceta.2011.12.004 22402235

[18] Bosch XB. Empleo precario y conflictos jurídicos en las administraciones públicas locales y autonómicas. In: Cuadernos Constitucionales de la Cátedra Fadrique Furió Ceriol. (2005). p. 52–3; 185–219. Available online at: https://dialnet.unirioja.es/servlet/articulo?codigo=2538799

[19] Pastor AJ. Un sistema sanitario contra un virus. Emergencias. (2020) 32:152–4. Available online at: http://emergencias.portalsemes.org/descargar/un-sistema-sanitario-contra-un-virus/32395919

[20] Sánchez-Bayón A. Derecho Público General: Instituciones Públicas y Actores Sociales. Madrid: Delta Publicaciones (2016).

[21] Sierra Moros MJ Monge S Suarez Rodríguez B García San Miguel L Simón Soria F. COVID-19 in Spain: view from the eye of the storm. Lancet Publ Health. (2021) 6:E10. 10.1016/S2468-2667(20)30286-3 33301724 PMC7833953

[22] Editorial-Lancet. COVID-19 in Spain: a predictable storm? Lancet Publ Health. (2020) 5:E568. 10.1016/S2468-2667(20)30239-5 33075295 PMC7567525

[23] Bagus P Peña-Ramos JA Sánchez-Bayón A. COVID-19 and the political economy of mass hysteria. Int J Environ Res Publ Health. (2021) 18:1–15. 10.3390/ijerph18041376 33546144 PMC7913136

[24] Sanchez-Bayon A Bagus P Pena-Ramos JA. Political economy of mass neurosis during the COVID-19 Crisis. J Infect Dis Res. (2021) 4:1–15. Available online at: https://www.scitcentral.com/article/62/2278/Political-Economy-of-Mass-Neurosis-during-the-COVID-19-Crisis 10.3390/ijerph18041376 PMC7913136 33546144

[25] Hayek FA (ed.). The nature and history of the problem. In: Collectivist Economic Planning: Critical Studies on the Possibilities of Socialism. London: Routledge (1935). p. 1–40.

[26] Hayek FA. The Road to Serfdom. London: George Routledge and Sons (1939).

[27] Hoppe H. A Theory of Socialism and Capitalism. Boston, MA: Kluwer. 10.1007/978-94-015-7849-3

[28] Hoppe H. The economics and ethics of private property (reprinted 2006). Auburn: Ludwig von Mises Institute (1993). 10.1007/978-94-015-8155-4

[29] Rothbard MN. The end of socialism and the calculation debate revisited. Rev Austr Econ. (1991) 5:51–76. 10.1007/BF02426928

[30] Boettke P. Socialism and the Market: The Socialist Calculation Debate Re-visited. London: Routledge (2000).

[31] Huerta de Soto J. Socialism, Economic Calculation and Entrepreneurship. Cheltenham: Edward Elgar Publishing Ltd. (2010). 10.4337/9781849805001

[32] Arnedo EG Valero-Matas JA Sánchez-Bayón A. Spanish tourist sector sustainability: recovery plan, green jobs and wellbeing opportunity. Sustainability. (2021) 13:11447. 10.3390/su132011447

[33] Sánchez-Bayón A García-Vaquero M Lominchar J. Wellbeing economics: beyond the labour compliance and challenge for business culture. J Leg Ethical Regul Issues. (2021) 24:1–15. Available online at: https://www.abacademies.org/articles/wellbeing-economics-beyond-the-labour-compliance–challenge-for-business-culture-11594.html

[34] Sánchez-Bayón A. Balance del sector público y su derecho orgánico y protocolario I: juridicidad de la matriz estatal y del desarrollo del Poder ejecutivo. Derecho Cambio Soc. (2020) 62:1–28. Available online at: https://www.derechoycambiosocial.com/revista062/Balance_del_Sector_publico.pdf

[35] Brennan G Buchanan J. The reason of rules: Constitutional Political Economy. Cambridge: Cambridge University Press (1985). 10.1017/CBO9780511895937

[36] Rey del Castillo J. Análisis del origen, situación y perspectivas de futuro del proceso de descentralización sanitario español. Rev Españ Salud Públ. (1998) 72:13–24. 10.1590/S1135-57271998000100003 9477712

[37] Gómez AR. La descentralización sanitaria en España: el camino recorrido y las tareas pendientes. Papeles de Economí*a Española*. (1998) 76:49–66. Available online at: https://www.funcas.es/publicaciones/revistas/papeles-de-economia/listado-papeles-de-economia/

[38] López-Casasnovas G Rico A. La descentralización, ‘parte del problema sanitario o de su solución? Gaceta Sanitar. (2003) 17:319–26. 10.1016/S0213-9111(03)71755-5 12975058

[39] Sanz IA. Evolución de la descentralización y el gasto público en España. Rev Española Control Externo. (2009) 11:77–106. Available online at: https://www.tcu.es/repositorio/2d0d14fe-4e6a-458d-8cc3-7e646731c57d/Revista%2031.pdf

[40] Sáenz Royo E. La prestación sanitaria en el Estado autonómico: las incongruencias entre el modelo competencial y su financiación. Rev Española Derecho Constit. (2020) 119:119–49. 10.18042/cepc/redc.119.04

[41] Gallego R. Descentralización y Desigualdad en el Estado Autonómico. Valencia: Tirant lo Blanch (2016).

[42] Grau NC. La transparencia en la gestión pública: Cómo construirle viabilidad? Estado Gobierno Gestión Públ Rev Chilena Administr Públ. (2006) 8:22–44. Available online at: https://dialnet.unirioja.es/servlet/articulo?codigo=2315250

[43] Meneu R Ortún V. Transparencia y buen gobierno en sanidad: también para salir de la crisis. Gaceta Sanitar. (2011) 25:333–8. 10.1016/j.gaceta.2011.02.010 21543139

[44] Bennett R Kottasz R. Practitioner perceptions of corporate reputation: an empirical investigation. Corpor Commun. (2000) 5:224–35. 10.1108/13563280010357349

[45] Mira JJ Lorenzo S Navarro IM Guilabert M Pérez-Jover V. La reputación de los hospitales españoles: bases para el desarrollo de un índice de reputación de los hospitals. Anal Sist Sanitar Navarra. (2015) 38:247–54. 10.4321/S1137-66272015000200008 26486530

[46] Pérez-Romero C Ortega-Díaz MI Ocaña-Riola R Martín-Martín JJ. Análisis de la eficiencia técnica en los hospitales del Sistema Nacional de Salud español. Gaceta Sanitar. (2017) 31:108–15. 10.1016/j.gaceta.2016.10.007 28043697

[47] Gost Garde J. Difusión de los resultados en salud de los hospitales. Anal Sist Sanitar Navar. (2015) 38:181–4. 10.4321/S1137-66272015000200001 26486523

[48] Elola J Nieto J Sunyer J Daponte A. La relación entre ideología y eficiencia de los sistemas sanitarios. Unas notas de cara a la reforma del sistema sanitario español. Gaceta Sanitar. (1996) 10:191–6. 10.1016/S0213-9111(96)71895-2 9081919

[49] Navarro IM Mira JJ Lorenzo S. Desarrollo y validación de un cuestionario para medir la reputación de los hospitales. Gaceta Sanitar. (2012) 26:444–9. 10.1016/j.gaceta.2011.11.020 22475812

[50] Asenjo MA Bertrán MJ Guinovart C Llach M Prat A Trilla A. Análisis de la reputación de los hospitales españoles: relación con su producción científica en cuatro especialidades. Med Clí*n*. (2006) 126:768–70. 10.1157/13089112 16792980

[51] Sánchez-Bayón A. A critical history of industrial sociology: from blue and white collar workers to Knowmads and freeriders. Miscelánea Comillas. (2019) 77:431–51. 10.14422/mis.v77.i151.y2019.008

[52] Sánchez-Bayón A. Renewal of business and economic thought after the globalization: talentism and happiness economics. Bajo Palab. (2020) 24:293–318. 10.15366/bp.2020.24.015

[53] Sánchez-Bayón A. A history of HR and its digital transformation. Rev Asoc Española Especial Med Trabajo. (2020) 29:198–214. Available online at: https://scielo.isciii.es/pdf/medtra/v29n3/1132-6255-medtra-29-03-198.pdf

[54] Sánchez-Bayón A Trincado E. Business and labour culture changes in digital paradigm. Cogito. (2020) 12:225–43. Available online at: http://cogito.ucdc.ro/COGITO%20septembrie%202020.pdf

[55] Sánchez-Bayón A Lominchar J. Labour relations development until the digital transition. J Legal Ethic Regul Issues. (2020) 23:1–13. Available online at: https://www.abacademies.org/articles/Labour-relations-development-until-the-digital-transition-1544-0044-23-6-568.pdf

[56] Menger C. Principles of Economics (Reprinted 2007). Auburn: Ludwing von Mises Institute (1871).

[57] Mises L. Epistemological Problems of Economics (reprinted 2003). Auburn: Ludwig von Mises Institute (1933).

[58] Mises L. The Ultimate Foundation of Economic Science. New York, NY: Van Nostrand (1962).

[59] Hayek FA. The Counter-Revolution of Science: Studies on the Abuse of Reason. Blencoe: Free Press (1952).

[60] Hayek FA. The theory of complex phenomena. In: Bunge M editor. Critical Approaches to Science and Philosophy. Boca Ratón, FL: CRC Press (1999). p. 332–49. 10.4324/9781351313087-22

[61] Hoppe H. Economic Science and the Austrian Method. Auburn: Ludwig von Mises Institute (1995).

[62] Daumann F Follert F. Learning from crises? Some philosophical and politico-economic considerations in the light of the COVID-19 pandemic. Proc Mercado Rev Eur Econ Polí*t*. (2021) 18:245–74. 10.52195/pm.v18i1.711

[63] Gleißner W Follert F Daumann F Leibbrand F. EU's ordering of COVID-19 vaccine doses: political decision-making under uncertainty. Int J Environ Res Public Health. (2021) 18:2169. 10.3390/ijerph18042169 33672157 PMC7926704

[64] Pérez-Calle RD Garcia-Casarejos N Garcia-Bernal J. La empresa española ante la COVID-19: factores de adaptación al nuevo escenario. Retos Rev Cienc Administr Econ. (2021) 11:5–24. 10.17163/ret.n21.2021.01

[65] Dyntra. Spanish Healthcare System. (2021). Available online at: https://www.dyntra.org/indices/servicios-sanitarios/ (accessed May 15, 2021).

[66] Merco. Merco's Healthcare Reputation Monitor-MRS. (2019). Available online at: https://www.merco.info/es/monitor-reputacionsanitaria-hospitales (accessed on May 15, 2021).

[67] CIS. Study No 3259 (in Oct. 2019, Before the Pandemic Warning): Sample of 2,464 Interviews and a Margin Error of ±2.0%. (2020). Available online at: http://www.cis.es/cis/opencm/ES/2_bancodatos/estudios/ver.jsp?estudio=14464andcuestionario=17441andmuestra=24405 (accessed May 15, 2021).

[68] CIS. Study No 3290 (in July 2020, During the pandemic second wave): sample of 2,926 interviews and a Margin Error of ±1.8%. (2020). Available online at: http://www.cis.es/cis/opencm/ES/2_bancodatos/estudios/ver.jsp?estudio=14517andcuestionario=17489andmuestra=24760 (accessed May 15, 2021).

[69] CIS. Study No 3285 (June 2020): With a Simple of 937 Interviews and a Margin Error of ±3.3%. (2020). Available online at: http://www.cis.es/cis/opencm/ES/2_bancodatos/estudios/ver.jsp?estudio=14512andcuestionario=17484andmuestra=24753 (accessed May 15, 2021).

[70] CIS. Study No 3298 (Oct. 2020): With a Simple of 2,861 Interviews and a Margin Error of ±1.9%. (2020). Available online at: http://www.cis.es/cis/opencm/ES/2_bancodatos/estudios/ver.jsp?estudio=14530andcuestionario=17506andmuestra=24838 (accessed May 15, 2021).

[71] CIS. Study No. 3277 (March, 2020): With a Simple of 3,911 Interviews and a Margin Error of ±1.6%. (2020). Available online at: http://www.cis.es/cis/opencm/ES/2_bancodatos/estudios/ver.jsp?estudio=14493andcuestionario=17472andmuestra=24722 (accessed May 15, 2021).

[72] CIS. Study No. 3305 (Dec. 2020): A Simple of 2,084 Interviews and a Margin Error of ±2.2%. (2020). Available online at: http://www.cis.es/cis/opencm/ES/2_bancodatos/estudios/ver.jsp?estudio=14538andcuestionario=17514andmuestra=24853 (accessed May 15, 2021).

[73] CIS. Study No. 3281 (May 2020), With a Sample of 3,800 Interviews and a Margin Error of ±1.6%. (2020). Available online at: http://www.cis.es/cis/opencm/ES/2_bancodatos/estudios/ver.jsp?estudio=14508andcuestionario=17480andmuestra=24728 (accessed May 15, 2021).

[74] CIS. Study No. 3,303 (Dec. 2020), With a Sample of 3,817 Interviews and a Margin Error of ±1.6%. (2020). Available online at: http://www.cis.es/cis/opencm/ES/2_bancodatos/estudios/ver.jsp?estudio=14536andcuestionario=17512andmuestra=24847 (accessed May 15, 2021).

